# Use of global coronary heart disease risk assessment in practice: a cross-sectional survey of a sample of U.S. physicians

**DOI:** 10.1186/1472-6963-12-20

**Published:** 2012-01-24

**Authors:** Benjamin Shillinglaw, Anthony J Viera, Teresa Edwards, Ross Simpson, Stacey L Sheridan

**Affiliations:** 1 Department of Medicine, University of Colorado Denver School of Medicine, Denver, Colorado, USA. At the time of writing, Dr. Shillinglaw was a student at the University of North Carolina at Chapel Hill School of Medicine and the Public Health Leadership Program of the Gillings School of Global Public Health, Chapel Hill, North Carolina, USA; 2 Department of Family Medicine, University of North Carolina at Chapel Hill School of Medicine, Chapel Hill, North Carolina, USA; 3 Odum Institute for Research in Social Science, University of North Carolina at Chapel Hill, Chapel Hill, North Carolina, USA; 4 Department of Medicine, Division of Cardiology, University of North Carolina at Chapel Hill School of Medicine, Chapel Hill, North Carolina, USA; 5 Department of Medicine, Division of General Medicine and Clinical Epidemiology, University of North Carolina at Chapel Hill School of Medicine, Chapel Hill, North Carolina, USA

## Abstract

**Background:**

Global coronary heart disease (CHD) risk assessment is recommended to guide primary preventive pharmacotherapy. However, little is known about physicians' understanding and use of global CHD risk assessment. Our objective was to examine US physicians' awareness, use, and attitudes regarding global CHD risk assessment in clinical practice, and how these vary by provider specialty.

**Methods:**

Using a web-based survey of US family physicians, general internists, and cardiologists, we examined awareness of tools available to calculate CHD risk, method and use of CHD risk assessment, attitudes towards CHD risk assessment, and frequency of using CHD risk assessment to guide recommendations of aspirin, lipid-lowering and blood pressure (BP) lowering therapies for primary prevention. Characteristics of physicians indicating they use CHD risk assessments were compared in unadjusted and adjusted analyses.

**Results:**

A total of 952 physicians completed the questionnaire, with 92% reporting awareness of tools available to calculate CHD global risk. Among those aware of such tools, over 80% agreed that CHD risk calculation is useful, improves patient care, and leads to better decisions about recommending preventive therapies. However, only 41% use CHD risk assessment in practice. The most commonly reported barrier to CHD risk assessment is that it is too time consuming. Among respondents who calculate global CHD risk, 69% indicated they use it to guide lipid lowering therapy recommendations; 54% use it to guide aspirin therapy recommendations; and 48% use it to guide BP lowering therapy. Only 40% of respondents who use global CHD risk routinely tell patients their risk. Use of a personal digital assistant or smart phone was associated with reported use of CHD risk assessment (adjusted OR 1.58; 95% CI 1.17-2.12).

**Conclusions:**

Reported awareness of tools to calculate global CHD risk appears high, but the majority of physicians in this sample do not use CHD risk assessments in practice. A minority of physicians in this sample use global CHD risk to guide prescription decisions or to motivate patients. Educational interventions and system improvements to improve physicians' effective use of global CHD risk assessment should be developed and tested.

## Background

Coronary heart disease (CHD) is the major killer of American men and women, responsible for one out of every six deaths in the United States (US) [1]. Remarkably, CHD is also largely preventable. According to a study of 52 countries, nine easily measured and potentially modifiable risk factors account for over 90% of the risk of an initial acute myocardial infarction [2]. Primary prevention of CHD should be a top priority due to the high rate of first events that are fatal, disabling, or that require expensive management [3]. Unfortunately, the majority of individuals with elevated CHD risk factors are not using appropriate risk reducing therapies [4-7]. One contributing factor is that clinicians often do not accurately estimate a patient's risk for CHD [8-12].

The risk for the development of CHD varies greatly among individuals. Effective clinical primary prevention of CHD therefore requires individualized interventions that range in intensity. In order to appropriately select medical interventions for primary prevention it is necessary to stratify patients based on an assessment of cardiovascular risk [13]. Current guidelines suggest that all patients ≥ 40 years of age or those with ≥ 2 risk factors should have their 10-year risk of CHD assessed every 5 years or as risk factors change, with a global risk assessment [3]. The risk factors used to calculate global CHD risk include: age, sex, smoking status, blood pressure (BP), total cholesterol (sometimes low density lipoprotein (LDL)), high density lipoprotein (HDL) cholesterol, and in some risk tools, diabetes [3]. There are many user-friendly, easily accessible tools available for estimating a patient's CHD risk including risk charts and risk calculators for personal digital assistants, personal computers, and web-based use [14]. When compared to the full Framingham equations for identifying patients at increased risk, these tools are generally accurate, although Framingham-based estimates may not apply equally to all ethnic groups [3,14].

Calculation of a patient's risk for CHD has many advantages in clinical practice. It allows improved prediction of incident events, enabling physicians to better identify patients who warrant preventive medications [15]. Use of global risk may also improve intermediate and long-term outcomes for patients, particularly when combined with counseling [15,16]. Patients may gain an improved understanding of their risk and the reason for any proposed interventions, which may increase motivation to adhere to any preventive medications that are prescribed [15,16].

While advantages of using global risk assessment in the primary prevention of CHD have been documented, little is known about the actual use of global CHD risk in clinical practice. The purpose of this study was to examine US physicians' awareness, attitudes towards CHD risk assessment, and use of CHD risk assessment when considering clinical interventions for primary prevention, and whether these vary across three relevant clinical specialties.

## Methods

### Overall design

This study was a cross-sectional, web-based survey of a national sample of family physicians, general internists, and cardiologists. The survey was designed by the investigators, pretested among a convenience sample of family physicians, general internists, and cardiologists, and automated by survey experts at the Odum Institute for Research in Social Science affiliated with the University of North Carolina at Chapel Hill. This study was approved by the Office of Human Research Ethics at the University of North Carolina at Chapel Hill.

### Study sample and invitations to participate

The sampling frame was family physicians who are members of the American Academy of Family Physicians (AAFP) and general internists and cardiologists who are members of the American College of Physicians (ACP). A mailing list of 9000 members randomly selected from a database of members of the AAFP (family physicians) and the ACP (general internists and cardiologists) was obtained. The list consisted of 2623 family physicians (377 members were excluded because they were medical students), 3000 general internists, and 3000 cardiologists.

Personalized letters of invitation were mailed to the 8623 physicians. These letters described the study and provided a URL for the online survey with an individualized identification code to allow tracking of non-responders. At two and four weeks after the initial invitation was sent out, non-respondents were mailed reminder letters. As an incentive to participate, physicians who wished to do so could have their name entered into a drawing for one of two $500 gift cards.

### Variables

Data obtained from the survey included physicians' awareness of tools available to calculate CHD risk, frequency of using global CHD risk in clinical practice, method of CHD risk assessment, and how often CHD risk calculations are used to guide prescription of aspirin, lipid-lowering, and BP lowering therapies for primary prevention (Appendix). We collected data on attitudes regarding the usefulness of CHD risk calculations and, among those who reported not using global CHD risk, reasons for not doing so. Information regarding respondents' specialty type, sex, year of graduation from medical school, amount of patient care time, type of practice setting, region of the country, use of electronic medical records and computers in exam rooms, and use of a personal digital assistant (PDA) or smart phone during patient encounters was also collected.

### Analysis

Responses to each item were tabulated, missing responses were excluded, and differences were compared by respondent characteristics. Testing for significant differences was performed using analysis of variance (ANOVA) for Likert-scaled outcomes, chi-square for categorical outcomes, and Kruskal-Wallis for ordinal outcomes. Attitudes towards CHD risk assessment were examined among physicians who reported being aware of CHD risk assessment using five statements. Physicians responding, "Strongly Agree", or Agree" were combined into an "Agree" category, while subjects who responded, "Disagree" or "Strongly Disagree" were combined into a "Disagree" category. (There was no "Neutral" category.) Proportions agreeing with each statement were tabulated and compared between specialty groups. Physicians who indicated that they were aware of and, "occasionally", "most of the time", or "always" obtain a calculation of a patients global CHD risk for primary prevention (based on the question, "When considering primary prevention of coronary heart disease in adults, how often do you obtain a calculation of a patient's global (overall) coronary heart disease risk?") were combined into a "CHD user" category. Those who indicated they were aware of but "never" or "rarely" calculated a patient's global CHD risk for primary prevention based on this question were combined into a "non-CHD user" category. Characteristics of the respondents in each category were compared in unadjusted analyses and then by logistic regression initially adjusting for specialty, years in practice, amount of patient care time, office practice setting, use of EMR, use of internet in patient exam rooms, sex, region of the country, and PDA use. Variables with an associated p-value > 0.10 were then excluded from a final model.

Among the "non-CHD user" group, potential reasons for not calculating patient's global CHD risk for primary prevention were examined through responses to six statements that were rated for importance on a Likert-scale from 0-5 (with 0 being not important at all- 5 being extremely important). The ratings were averaged for each statement and compared in unadjusted analyses by specialty.

To examine physicians' use of CHD risk to guide primary preventive pharmacological therapy, respondents indicating that they are aware of and use CHD risk estimates to guide recommendations, "occasionally", "most of the time", or "always or nearly always", were categorized as those who use CHD risk to guide preventive therapy and were compared to those who rarely or never use such calculations. Results were also compared by specialty.

Statistically significant differences were defined as a p-value < 0.05. To assess the potential for nonresponse bias, geographic regions between respondents and non-respondents were compared. All analyses were performed using Stata 10.1 statistical software (StataCorp, College Station, TX).

## Results

### Study participants

Of the 8623 letters mailed, 74 were returned as undeliverable, including 8 because the intended recipient was deceased, and 3 because of delivery refusal. A total of 1238 physicians participated in the survey. Respondents who indicated they did not see patients in the office setting (n = 251) or whose practicing specialty was one other than family medicine, general internal medicine, or cardiology (n = 55) were excluded. The adjusted response rate was 15%. The final sample consisted of 952 physicians: 390 (41%) family physicians, 272 (29%) general internists, and 290 (30%) cardiologists (Table 1). A total of 932 (98%) of respondents completed every survey item, while 20 (2%) submitted partial responses. Nonrespondents were compared to respondents by geographic region, and the two groups were very similar (Additional File 1).

**Table 1:** Characteristics of respondents (N = 952)

	All	Family physicians (n = 390)	General Internists (n = 272)	Cardiologists (n = 290)	P- value*
	%	%	%	%	

% Male	73.6	57.8	74.6	94.3	< 0.001

Years in Practice					< 0.001

> 20	63.1	40.2	70.2	88.0	
10-19	18.8	26.0	19.9	8.0	
< 10	18.1	33.9	9.9	4.0	

Region of country					0.003

Northeast	23.7	18.3	25.7	29.3	
South	33.2	34.3	33.6	31.4	
Midwest	24.2	24.5	24.2	23.9	
West	18.9	22.9	16.6	15.4	

Time spent in office based care					< 0.001

> 75%	56.5	71.0	63.5	30.3	
51-74%	14.7	9.5	11.1	25.1	
50%	8.5	5.6	6.6	14.3	
25-49%	10.2	9.0	6.6	15.3	
< 25%	10.0	4.9	12.2	15.0	

Practice setting					< 0.001

Solo Practice	12.5	9.7	14.8	14.1	
Small Group (2-9 clinicians)	32.0	36.7	32.6	25.2	
Large Single Specialty group (10+ clinicians)	11.8	9.7	4.8	21.0	
Large multi-specialty group (10+ clinicians)	13.9	13.9	18.5	9.7	
Academic group	20.6	21.8	17.0	22.4	
Other	9.2	8.2	12.2	7.6	

Use of electronic medical records	59.2	59.4	58.2	59.9	0.91

Computers in exam rooms	55.5	56.8	54.1	55.2	0.78

Internet connection for computers in exam rooms	94.1	92.7	95.9	94.3	0.46

Use personal digital assistant or smartphone	47.4	61.5	36.9	38.1	< 0.001

*Overall P-values based on Pearson's Chi square test of significance or Kruskal-Wallis test between specialty groups

Of those who responded, the majority were male (74%), spent more than 75% of work time in office based patient care (56%), use EMR in their practices (59%), have computers in patient exam rooms (56%), and have been in practice for 10 years or more (79%). Small group practices were the most common practice setting (32%), and the most common region of the country practiced in was the South (33%).

Across provider respondent groups, there were some notable differences. Cardiologists (94%) who responded were more likely than general internists (75%) and family physicians (58%) to be male. Cardiologists (88%) were more likely than general internists (70%) or family physicians (40%) to have been in practice for more than 20 years. General internists and family physicians spent more time in office-based care than cardiologists. Family physicians (62%) were more likely than general internists (37%) and cardiologists (38%) to use a PDA or smartphone when seeing patients in the office.

### Awareness and use of tools to calculate global CHD risk

Of all physicians (N = 951) who responded to the question, "Have you heard about tools to calculate a patients overall risk of coronary heart disease in the next 10 years (global CHD risk)?" 873 responded "yes" (92%). When those in this group were asked, "When considering primary prevention of CHD in adults, how often do you obtain a calculation of a patient's global (overall) coronary heart disease risk?", a total of 358 respondents (41%) reported using global CHD risk at least occasionally when considering primary prevention of CHD in adults, including 67 (8%) who reported always or nearly always doing so. The remaining 505 physicians (59%) who responded to this question (10 responses were missing) reported that they "rarely" or "never" use global CHD risk assessment when considering primary prevention of CHD in adults. One third of respondents (33%) who use global CHD risk in practice reported using a web-based application, while 27% use a paper chart and 26% use a program on a PDA or smartphone to obtain their patient's risk estimate (Table 2). Few (14%) use other methods such as non-web based computer programs (e.g., a spreadsheet) or calculators embedded in EMRs.

**Table 2:** Reported method of CHD global risk assessment among physicians who use CHD risk assessment (N = 358)

	All, %	Family physicians, %	General internists, %	Cardiologists, %	P- Value*
Paper chart (n = 96)	27.0	22.8	25.7	32.3	0.18

Web-based (n = 117)	32.9	29.5	40.5	32.3	0.12

Non-web-based computer program (n = 23)	6.2	3.4	8.1	8.3	0.01

Program on a personal digital assistant (n = 94)	26.1	34.9	18.9	20.3	0.02

Other (n = 28)	7.9	9.4	6.8	6.8	0.09

* Overall P-value based on Pearson's Chi square test for significance between specialty groups

The majority of respondents who indicated they were aware of CHD risk assessment tools (with no significant difference between specialties) agreed or strongly agreed that global CHD risk calculation is useful, improves patient care, leads to better decisions about whether or not to recommend therapies to prevent heart disease events, and increases the likelihood that they will recommend risk reducing therapies to prevent heart disease (Table 3). However, a minority who indicated they were aware of CHD risk assessment tools reported actually using global CHD risk to guide their primary preventive pharmacotherapy recommendations.

**Table 3:** Percentage of respondents aware of CHD risk assessment tools who agree with the given statements regarding global CHD risk assessment (N = 873)

	All	Family Physicians	General Internists	Cardiologists	P- value*
	**%**	**%**	**%**	**%**	

I find global CHD risk calculation useful (n = 834)	83.8	85.6	80.9	84.0	0.33

Global CHD risk calculation wastes time (n = 811)	18.7	17.1	18.3	21.4	0.40

Global CHD risk calculation improves patient care (n = 825)	80.9	82.8	80.6	78.5	0.41

Global CHD risk calculation leads to better decisions about whether or not to recommend therapies to prevent heart disease events (n = 819)	81.1	82.8	82.8	77.3	0.19

Global CHD risk calculation increases the likelihood that I will recommend risk-reducing therapies to prevent heart disease (n = 809)	71.2	73.5	73.7	65.9	0.08

CHD = coronary heart disease

*Overall p-values based on Pearson's chi squared test for significance between specialty groups

Approximately 69% of respondents who calculate global CHD risk use it to guide lipid lowering therapy recommendations; 54% use it to guide aspirin therapy recommendations; and 48% use it to guide BP lowering therapy recommendations (Table 4). Approximately 40% of respondents who use CHD risk calculation reported that they tell their patients his/her CHD risk estimation "most of the time", "nearly always", or "always". Cardiologists were more likely to report that they tell a patient his/her CHD risk estimation (49%) than were family physicians and general internists (38% and 33% respectively) (p < 0.01).

**Table 4:** Physicians' reports of how they use CHD global risk assessment for primary prevention, among those who use CHD risk assessment (N = 358)

	All	Family Physicians	General Internists	Cardiologists	P- value*
	**%**	**%**	**%**	**%**	

Use global CHD risk estimate to guide lipid lowering therapy recommendations (n = 355)	69.0	63.5	72.4	73.3	0.16

Use global CHD risk estimate to guide aspirin therapy recommendations (n = 350)	53.7	52.4	51.4	56.5	0.72

Use global CHD risk estimate to guide blood pressure lowering therapy recommendations (n = 356)	48.3	48.7	44.7	50.0	0.76

Use global CHD risk estimate to guide any primary prevention therapy recommendation (n = 358) †	76.8	73.2	79.0	79.7	0.38

Tell patients their global CHD risk (n = 352)	40.1	37.7	33.0	49.2	0.01

CHD = coronary heart disease

*Overall p-values based on Pearson's chi squared test for significance between specialty groups

† Based on using risk estimate to guide lipid lowering, aspirin, or blood pressure lowering recommendation

### Physicians who use global CHD risk assessment

Among physicians who reported being aware of global CHD risk assessment tools, reports of using global CHD risk assessment differed by specialty, years in practice, time spent in office-based care, and use of a PDA or smartphone (Table 5). Among cardiologists, 49% reported using global CHD risk assessment, compared to approximately 42% of family physicians and 32% of general internists (p < 0.001). Those who had been in practice for 10-19 years (50%) were more likely than those who have been in practice for less than 10 (41%) and more than 20 years (39%) to use CHD risk assessment (p = 0.02). Respondents who indicated they used a PDA or smartphone when seeing patients were more likely to report using global CHD risk assessment (47% vs. 36%, p = 0.001). There were no differences in use of global CHD risk assessment among those who used EMRs or those who had computers/internet connection in exam rooms. The final multivariate logistic regression model showed that after adjusting for specialty, years in practice, office-based care time, and PDA or smart phone use, physicians who reported using a PDA or smartphone had greater odds of using CHD risk assessment in practice (OR 1.58; 95% CI 1.17-2.12) (Table 6). Cardiologists, physicians in practice 10-19 years, and those spending either 50% time or 51-74% time in office based care also had greater odds of being users.

**Table 5:** Proportion of physicians who are aware of and report using CHD risk assessment, by subgroups of physician characteristics (N = 873)

	%	P- value*
All	41.4	

Specialty		< 0.001†

Family medicine	41.7	
Internal medicine	31.9	
Cardiology	49.4	

Years in practice		0.02

> 20	38.9	
10-19	49.7	
< 10	41.3	

Sex		0.92

Male	41.6	
Female	41.2	

Region of country		0.48

Northeast	44.0	
Southeast	42.0	
Midwest	37.0	
West/West coast	43.1	

Time spent in Office based patient care		0.002‡

> 75%	36.3	
51-74%	50.8	
50%	57.5	
25-49%	43.7	
< 25%	40	

Office Setting		0.37

Solo practice	39.4	
Small group	42.1	
Large Single Specialty	44.2	
Large Multi-specialty	33.1	
Academic Group	45.9	
Other	41.1	

Use electronic medical records		0.71

Yes	40.9	
No	42.2	

Computers in Exam Rooms		0.67

Yes	42.0	
No	40.6	

Internet Connection available for computers in Exam Rooms		0.53

Yes	42.5	
No	36.7	

Use a PDA or smartphone when seeing patients		0.001

Yes	47.1	
No	36.2	

CHD = coronary heart disease; PDA = personal digital assistant

*Overall P-values based on Pearson's Chi square

†Pearson's Chi square tests between paired groups yielded p = 0 .072 between cardiologists and family medicine physicians, p < 0 .0001 between cardiologists and general internists, and p = 0.018 between family medicine physicians and general internists.

‡ Pearson's Chi square tests between paired groups yielded p < 0 .003 for respondents spending > 75% of time vs. those spending 51-74% of time, p < 0 .001 for > 75% vs. 50%, p < 0.03 for 50% of time vs. < 25%.

**Table 6:** Odds of using CHD risk assessment by subgroups of physician characteristics

Characteristic	Odds Ratio (95% CI)*
Specialty	
Internal medicine	1.0 (referent)
Cardiology	1.95 (1.31 - 2.90)
Family medicine	1.36 (0.93 - 1.98)

Years in practice	
< 10 years	1.0 (referent)
10-19 years	1.70 (1.06 - 2.73)
≥20 years	0.93 (0.61 - 1.42)

Office based care time	
≥75%	1.0 (referent)
51-74%	1.61 (1.05 - 2.46)
50%	2.25 (1.33 - 3.82)
25-49%	1.21 (0.74 - 1.97)
< 25%	1.19 (0.72 - 1.57)

PDA or smartphone use	1.58 (1.17 - 2.12)

*Based on the results of a logistic regression model, adjusted for specialty, years in practice, amount of office based care time, and use of a PDA/smartphone.

### Reasons for not using global CHD risk assessment

Among physicians' who reported not using CHD risk assessment (N = 492; 505 minus 13 missing responses), the reason with the highest mean importance rating (2.6 ± 1.6) on a 0 to 5 scale was, "It is too time consuming" (Table 7). Family physicians (2.8) rated this reason higher than general internists (2.6) and cardiologists (2.3) (p = .02). The reason with the next highest mean importance rating was, "I do not find it useful in practice" (2.2 ± 1.6). Cardiologists (2.9) rated this reason higher than general internists (2.0) and family physicians (1.9) (p < .0001). Lack of familiarity with how to use the risk calculation and lack of easy-to-use tools were rated slightly less important, particularly by the family physician and general internist respondents. Lack of accurate tools and a perception that the risk calculation is not valid for [my] patients received ratings indicating they were the least important of the listed reasons.

**Table 7:** Mean ratings of importance of reasons for never or rarely calculating patients' global CHD risk, amongst 'non-users', rated 0-5* (N = 492)

	All	Family Physicians	General Internists	Cardiologists	P-value†
It is too time consuming	2.6	2.8	2.6	2.3	0.02

I do not find it useful in practice	2.2	1.9	2.0	2.9	< 0.0001

I am not familiar enough with how to use the risk calculation	2.0	2.3	2.3	1.3	< 0.0001

There are no easy to use tools available for obtaining the calculation	1.8	2.0	1.8	1.5	0.01

There are no accurate tools available for obtaining the calculation	1.3	1.3	1.4	1.2	0.51

I do not think that the calculated heart disease risk is valid for my patient population	1.2	1.1	1.2	1.4	0.13

CHD = coronary heart disease

* "0" is the lowest importance level (not important at all), and "5" is the highest importance level (extremely important)

†Overall P-value based on analysis of variance test for significance between specialty groups

## Discussion

We sought to examine physicians' self-reported awareness, use in clinical practice, and attitudes regarding CHD global risk assessment for primary prevention and how this varies by provider type. Our study found that among a sample of US physicians: (1) awareness of tools to calculate CHD global risk is extremely high, (2) use of CHD global risk calculation in practice is low, (3) the most strongly endorsed reason for not calculating a patient's global CHD risk appears to be that it is too time consuming, (4) overall use of global CHD risk calculation to guide primary preventive pharmacologic therapy is low and infrequently used to guide aspirin recommendations.

Our finding that use of CHD global risk calculation by US physicians is low is concerning since national guidelines for primary prevention of CHD are based on individuals' calculated 10-year CHD risk levels. For example, use of global CHD risk is advocated by national cholesterol treatment guidelines to better identify people who will benefit from intensive treatment [17]. Additionally, the decision to use aspirin for primary prevention is one that needs to be weighed against the potential for harm from gastrointestinal bleeding or hemorrhagic stroke [18]. Due in part to the potential harms associated with preventive pharmacotherapy, calculating a patient's global CHD risk is an important step, allowing adjustment of the intensity of intervention to the overall risk:benefit ratio for the patient [3,13,17,18]. Still, in our sample, the majority of physicians who were aware of CHD risk assessment tools reported they did not use CHD risk assessment to guide primary preventive pharmacotherapy decisions. Evidence suggests that when 10-year coronary risk information is given to physicians, prescription of guideline concordant lipid-lowering and aspirin therapies is slightly improved [19].

The most commonly endorsed barrier to CHD risk assessment use is the perception that it is too time consuming. It has been shown previously that one of the main barriers to delivery of preventive health services in primary care is lack of time [20]. However, a recent study found that even with limited time, primary care physicians address many of the highest rated preventive services, including cholesterol and BP management, adequately [20]. While lack of time during primary care patient visits is certainly a valid concern, there are many tools available that offer quick and accurate calculation of a patient's CHD risk [14]. Physicians in our sample who use a PDA or similar device when seeing patients in the clinic were more likely to use CHD risk assessments than those who do not. This suggests that CHD risk calculator programs for PDAs or smartphones may be a method of increasing CHD risk assessments among physicians. Fortunately, several CHD risk calculation tools are already available for such devices [14]. Other CHD risk assessment tools include paper risk charts, spreadsheet programs for computers and web-based calculators [14]. Additionally, some EMR's extract risk factor data from patients' records and calculate and display 10-year CHD risk for clinical use. Uncertainty remains regarding which tool for calculating CHD risk produces the most favorable patient outcomes. One study showed that use of a computer based clinical support system added to a paper risk chart was not as effective as a paper chart alone in terms of systolic BP control over one year [21]. However, the computer based system required manual input of patient risk factors, as opposed to an automatic risk calculation embedded in an EMR [21].

Other barriers are that some physicians report that they do not find the assessment of global CHD risk useful in practice and are unsure how to use the risk calculation in practice. Cardiologists were most likely to indicate that CHD risk assessment is not useful in clinical practice, which could potentially be related to the lower proportion of patients seen for which primary prevention (rather than secondary prevention) is of concern. General internists and family physicians were more likely to indicate that they are not familiar enough with how to use CHD risk calculations, and that there are no accurate or easy tools available to calculate CHD risk. These responses suggest a need to develop educational interventions for physicians that discuss the use of global CHD risk calculations in clinical practice [2,3,22].

In addition to its usefulness in helping clinicians and patients make decisions about preventive pharmacotherapy that take into account the balance of benefits and harms, global CHD risk could also be used to motivate patients [15,16]. However, we found that only 40% of those who use CHD risk assessments inform patients of their global CHD risk estimate. In total, while a majority of physicians who use CHD risk assessments use them either to guide prescription decisions or to motivate patients, an appreciable number do not. This suggests that even when CHD risk is calculated by physicians in practice, they may be unaware of how to utilize this risk information to its full advantages.

Our findings illustrate the need for interventions to increase uptake and effective use of global CHD risk assessment for guiding primary prevention. Development of effective interventions to improve guideline adherence by physicians should consider the variety of barriers to implementation in order to be successful [23]. While the type of intervention that is most effective remains unknown, education in small doses as well as passive guideline dissemination have been shown to be ineffective methods for affecting physician behavior change [24]. Paper and electronic reminders may be the most effective single intervention; however, it is likely that multiple tools will be necessary to increase guideline adherence among physicians [24]. The use of guideline concordant decision aids, programs embedded in EMRs that automatically calculate and display risk values and action thresholds, and risk charts in patient exam rooms are examples of system improvements that warrant further investigation.

## Limitations

The major potential limitation of this study is that of non-response bias. If physicians who responded to our survey were more interested in cardiovascular disease prevention compared to those who did not respond, then our results may be biased. Physicians who are more passionate about primary prevention of CVD might be more likely to be aware of tools used to calculate global CHD risk and more likely to answer questions in agreement with the utility of global CHD risk calculation. The 92% awareness of tools to calculate global CHD risk suggests this may be the case. Thus, our results are likely overestimates of the awareness and perceived usefulness of CHD risk assessments.

Additionally, significantly more family physicians responded to our survey (41%) than did cardiologists (30%) or general internists (29%). This could have also contributed to overestimation of the awareness and perceived usefulness of CHD risk assessments as family physicians would be more likely than cardiologists to focus on primary prevention of CHD in practice.

Another potential limitation is that of sampling bias. If physicians who are members of the AAFP and ACP are different from physicians who choose not to be members, then our sample may not be reflective more generally of US physicians of the included specialties. Another clue that our sample may not be representative is the high use of EMRs by respondents. The web-based format of our questionnaire may have also selected for physicians who are more confident using computer based programs (including CHD risk assessment tools) and may potentially represent an overestimate of use of CHD global risk assessment compared to the general US physician population. Self-reported data is also difficult to interpret as physicians may have supplied socially desirable responses to survey items that differ from how they actually practice. Finally, the cross-sectional study design identifies associations but is insufficient to determine the cause of low CHD global risk assessment use amongst our sample.

## Conclusions

Among respondents to a survey about cardiovascular disease prevention, awareness of tools to calculate global CHD risk is extremely high; however, the majority of responding physicians do not use CHD risk assessments in practice. Use of PDAs in practice is associated with greater self-reported use of CHD risk assessment. One perceived barrier to using global risk calculation is that it is too time consuming. Many physicians who report using CHD risk calculation do not use it for guiding prescription decisions or patient motivation. Taken together, these findings suggest that educational interventions and system improvements are needed to improve physicians' use of global CHD risk to support primary preventive therapeutic decisions.

## Competing interests

The authors declare that they have no competing interests.

## Authors' contributions

AJV and SLS conceived of the study. BS conducted the analyses, drafted the initial manuscript and revisions of the manuscript. TE assisted with survey design and implementation and provided critical review of the manuscript. AJV assisted with interpreting data, drafting the initial manuscript, and drafting revisions. RS provided critical review and revisions to the manuscript. SLS provided critical input on the initial manuscript and revisions of the manuscript and assistance in interpreting data. All authors read and approved the final manuscript.

## Appendix

### Physician survey items

(1) Do you see patients in the office or other ambulatory care setting?

○ Yes

○ No

(2) Please indicate your specialty:

○ Cardiology

○ Family medicine

○ General internal medicine

○ Other (please specify)

(3) Approximately what percent of your work time is spent in office-based patient care?

○ 75% or more

○ 51-74%

○ 50%

○ 25-49%

○ Less than 25%

(4) Which of the following best describes your office practice setting?

○ Solo practice

○ Small group practice (2-9 clinicians)

○ Large single specialty group (10+ clinicians)

○ Large multi-specialty group (10+ clinicians)

○ Academic group practice

○ Other (please specify)

(5) Does your office practice use an electronic medical record?

○ Yes

○ No

(6) In your office practice, do you use computers in the exam rooms?

○ Yes

○ No

(7) Do the computers in the exam rooms have internet access?

○ Yes

○ No

(8) Do you use a personal digital assistant (e.g., Palm device, iPhone) when seeing patients in the office?

○ Yes

○ No

*This section is about global coronary heart disease (CHD) risk. An estimate of a patient's global (or overall) risk of having a coronary heart disease event can be made by combining his or her risk factors in an empirically-based equation using one of a variety of tools*.

(9) Have you heard about tools to calculate a patient's overall risk of having a coronary heart disease event in the next 10 years (global CHD risk)?

○ Yes

○ No

(10) In terms of 10-year coronary heart disease (CHD) risk, at what level of risk do you consider a patient to be "high risk" for CHD events?

○ 3% or above

○ 6% or above

○ 10% or above

○ 15% or above

○ 20% or above

○ 25% or above

○ 30% or above

○ 50% or above

(11) In terms of 10-year coronary heart disease (CHD) risk, below what level of risk do you consider a patient to be "low risk" for CHD events?

○ 3% or less

○ 6% or less

○ 10% or less

○ 15% or less

○ 20% or less

○ 25% or less

○ 30% or less

○ 50% or less

(12) <If aware> When considering primary prevention of coronary heart disease in adults, how often do you obtain a calculation of a patient's global (overall) coronary heart disease risk?

○ Never

○ Rarely (one to two out of every 10 adults seen for routine medical care)

○ Occasionally (three to five out every 10 adults seen for routine medical care)

○ Most of the time (six to eight out of every 10 adults seen for routine medical care)

○ Always or nearly always (nine to ten out of every 10 adults seen for routine medical care)

(13) <If answered "Never" or "Rarely" above> On a scale of 0 to 5, where 0 is not at all important and 5 is extremely important, rate the importance of each of the following reasons why you never or rarely obtain a calculation of a patient's global (overall) coronary heart disease risk.

○ I am not familiar enough with how to use the risk calculation

○ I do not find it useful in practice

○ There are no accurate tools available for obtaining the calculation

○ There are no easy to use tools available for obtaining the calculation

○ It is too time consuming

○ I do not think that the calculated heart disease risk is valid for my patient population

(14) <If user> Which one of the following do you most commonly use to obtain a patient's global (overall) coronary heart disease risk estimate?

○ A paper chart

○ A web-based application

○ A non-web-based computer program (e.g., spreadsheet calculator or personal computer)

○ A program on a personal digital assistant

○ Other (please specify)

(15) <If aware> Please indicate your level of agreement or disagreement (1 = strongly agree, 2 = agree, 3 = disagree, 4 = strongly disagree) with the following statements:

○ I find global CHD risk calculation useful.

○ Global CHD risk calculation improves patient care.

○ Global CHD risk calculation leads to better decisions about whether or not to recommend therapies to prevent heart disease events.

○ Global CHD risk calculation wastes time.

○ Global CHD risk calculation increases the likelihood that I will recommend risk-reducing therapies to high-risk patients to prevent heart disease.

(16) <If user> How often do you tell the patient his/her global (overall) coronary heart disease risk estimate?

○ Never

○ Rarely (one to two out of 10 adults for whom I calculate risk)

○ Occasionally (three to five out of 10 adults for whom I calculate risk)

○ Most of the time (six to eight out of 10 adults for whom I calculate risk)

○ Always or nearly always (nine to ten out of 10 adults for whom I calculate risk)

(17) <If user> Thinking about all the patients you are considering for primary prevention of coronary heart disease, how often do you use a global (overall) coronary heart disease risk estimate to guide your recommendations about lipid lowering therapy?

○ Never

○ Rarely (one to two out of 10 adults)

○ Occasionally (three to five out of 10 adults)

○ Most of the time (six to eight out of 10 adults)

○ Always or nearly always (nine to ten out of 10 adults)

(18) <If user> Thinking about all the patients you are considering for primary prevention of coronary heart disease, how often do you use a global (overall) coronary heart disease risk estimate to guide your recommendations about aspirin therapy?

○ Never

○ Rarely (one to two out of 10 adults)

○ Occasionally (three to five out of 10 adults)

○ Most of the time (six to eight out of 10 adults)

○ Always or nearly always (nine to ten out of 10 adults)

(19) <If user> Thinking about all the patients you are considering for primary prevention of coronary heart disease, how often do you use a global (overall) coronary heart disease risk estimate to guide your recommendations about blood pressure lowering therapy?

○ Never

○ Rarely (one to two out of 10 adults)

○ Occasionally (three to five out of 10 adults)

○ Most of the time (six to eight out of 10 adults)

○ Always or nearly always (nine to ten out of 10 adults)

(20) In what year did you graduate from medical school? (drop-down menu)

(21) Please indicate your sex.

○ Male

○ Female

(22) In which region of the country do you practice?

○ West

○ Midwest

○ South

○ Northeast

## Pre-publication history

The pre-publication history for this paper can be accessed here:

http://www.biomedcentral.com/1472-6963/12/20/prepub

## Supplementary Material

Additional file 1 **Geographic regions of respondents vs nonrespondents**. The table shows that the geographic regions between respondents and nonrespondents were similar. Click here for file
