# Supplementary material for: Use of global coronary heart disease risk assessment in practice: a cross-sectional survey of a sample of U.S. physicians
Source: BMC Health Serv Res. 2012 Jan 24;12:20. doi: 10.1186/1472-6963-12-20 (PMC3292915; doi:10.1186/1472-6963-12-20)
Supplement: Additional file 1 — Geographic regions of respondents vs nonrespondents. The table shows that the geographic regions between respondents and nonrespondents were similar. [file 1472-6963-12-20-S1.DOC]

**Table - Geographic regions of respondents vs nonrespondents**

|  | Respondents (N=987) | Nonrespondents (N=7312) |
| --- | --- | --- |
| West | 17.9% | 19.8% |
| Midwest | 23.9% | 22.8% |
| South | 34.7% | 33.9% |
| Northwest | 23.5% | 23.5% |
